# Participant Experiences in a Kidney Failure Care Intervention in the Navigate-Kidney Study

**DOI:** 10.1001/jamanetworkopen.2025.48506

**Published:** 2025-11-07

**Authors:** Katherine Rizzolo, Julie Ressalam, Kayla Robledo, Elizabeth Juarez-Colunga, Neil R. Powe, Jennifer E. Flythe, Russell E. Glasgow, Spero M. Manson, Romana Hasnain-Wynia, Michel Chonchol, Seth Furgeson, Allison Jaure, Chiadi E. Ndumele, Claudia Camacho, Daniel Cukor, Ladan Golestaneh, Delphine S. Tuot, Lauren McBeth, Lilia Cervantes

**Affiliations:** 1 Boston University Chobanian & Avedisian School of Medicine and Boston Medical Center, Section of Nephrology, Boston, Massachusetts; 2 Evans Center for Implementation and Improvement Sciences, Boston University Chobanian & Avedisian School of Medicine, Boston Massachusetts; 3 Department of Medicine, University of Colorado Anschutz Medical Campus, Aurora; 4 Department of Medicine, Priscilla Chan and Mark Zuckerberg San Francisco General Hospital, San Francisco, California; 5 Department of Medicine, University of California, San Francisco; 6 Division of Nephrology and Hypertension, Department of Medicine, University of North Carolina School of Medicine, Chapel Hill; 7 Cecil G. Sheps Center for Health Services Research, University of North Carolina, Chapel Hill; 8 Department of Family Medicine and ACCORDS Dissemination and Implementation Science Program, University of Colorado Anschutz Medical Campus, Aurora; 9 Centers for American Indian and Alaska Native Health, Colorado School of Public Health, University of Colorado Anschutz Medical Campus, Aurora; 10 Department of Renal Diseases and Hypertension, University of Colorado Anschutz Medical Campus, Aurora; 11 Sydney School of Public Health, The University of Sydney, Sydney, New South Wales, Australia; 12 Centre for Kidney Research, The Children’s Hospital at Westmead, Sydney, Australia; 13 Division of Cardiology, Department of Medicine, John Hopkins University School of Medicine, Baltimore, Maryland; 14 Departments of Medicine and Psychiatry, NYU Grossman School of Medicine, New York, New York; 15 Department of Medicine, Section of Nephrology, Yale School of Medicine, New Haven, Connecticut; 16 Division of Nephrology, Zuckerberg San Francisco General Hospital, University of California, San Francisco, San Francisco

## Abstract

**Importance:**

Latino individuals with dialysis-dependent kidney failure experience social challenges that render self-management of kidney disease difficult. In the Navigate-Kidney trial, a community health worker (CHW) intervention for Latinos receiving dialysis, intervention recipients experienced a decrease in interdialytic weight gain, a surrogate end point for patient self-management. Understanding how the Navigate-Kidney trial was experienced by participants is important when considering scalability and sustainment of the intervention.

**Objective:**

To examine participant perspectives on their experience in the Navigate-Kidney intervention.

**Design, Setting, and Participants:**

This qualitative study used semistructured interviews with participants receiving the Navigate-Kidney intervention. Participants received care at dialysis centers in Denver, Colorado, between November 2020 and August 2022 and were randomized to receive the Navigate-Kidney intervention. Interview transcripts were deidentified and analyzed using deductive thematic analysis. Data were examined from September 2024 to July 2025.

**Main Outcomes and Measures:**

The main outcomes were the themes and subthemes from the interviews.

**Results:**

Twenty-four Latino individuals (11 females [46%]; 13 males [54%]; mean [SD] age of 56 [11] years) participated. The identified themes and subthemes were (1) build trust through understanding health experience (cultivating a personalized connection, promoting optimism and resilience, demonstrating consistency and reliability); (2) address multilevel social and structural challenges to facilitate health system navigation (addressing health insurance barriers, improving food security, relieving the stress of administrative and financial tasks, bridging registered dietitian guidance, enabling attendance of and adherence to dialysis sessions and appointments, coordinating nonnephrology clinical care); (3) provide patient-centered education (improving language concordant communication, promoting health and numeracy literacy, providing culturally responsive dietary restriction); and (4) enhance self-management (exploring opportunities for transplant and peritoneal dialysis, facilitating quality of dialysis care and symptom management, encouraging medication adherence, increasing confidence in diet and fluid intake self-management, achieving clinical parameter targets, activating patients).

**Conclusions and Relevance:**

In this qualitative study of participants in the Navigate-Kidney trial, participants’ experience with CHWs built trusting relationships through empathy and consistent support, helped them navigate complex health and social systems, provided culturally and linguistically tailored education, and empowered them to manage their care. Understanding participant perspectives about those activities that promoted health and well-being is critical for future patient-centered adaptations of Navigate-Kidney and similar interventions.

## Key Points

**Question:**

How did community health workers (CHWs) in the Navigate-Kidney trial support patients receiving dialysis in managing their health and navigating the health care system?

**Findings:**

In this qualitative study of 24 Latino individuals, patients described that CHWs fostered trust through empathy and reliability, addressed social and logistical barriers to care, provided culturally and linguistically tailored health education, and enhanced confidence in self-management, medication adherence, and dialysis attendance.

**Meaning:**

These findings suggest that CHWs played a vital role in improving patient engagement, emotional well-being, and self-efficacy, suggesting their integration into dialysis care teams may enhance holistic, patient-centered care.

## Teaser

This qualitative study examines the perspectives of Latino individuals with dialysis-dependent kidney failure who received the Navigate-Kidney intervention.

## Introduction

Latino individuals with dialysis-dependent kidney failure face multilevel challenges to self-management including lack of culturally responsive care, food insecurity, low access to health insurance and specialty care, and low health literacy.^1,2,3,4^ These challenges can manifest in missed and shortened dialysis sessions, poorly controlled blood pressure, and higher interdialytic weight gain (IDWG, defined as accumulated fluid between dialysis sessions).^5,6,7^ Latino individuals receiving dialysis have described the dialysis diet restrictions and lack of patient-centered kidney care education as key barriers to self-management on dialysis.^1,8,9,10,11^ To meet the unique needs for this population, we developed Navigate-Kidney, a community health worker (CHW) intervention designed for individuals with kidney failure, to improve self-management on dialysis.^12^

Understanding how the intervention will fit into a diverse, dynamic, real-world setting is critical for ensuring intervention fit for the context. The Forms and Functions framework operationalizes 2 constructs that need to be considered when implementing an intervention: distinguishing the intervention’s core purposes (ie, functions) from the adaptable ways they are carried out (ie, forms).^12^ This framework can guide which elements must remain consistent, such as building trust or facilitating self-management, and which can be flexibly adapted (eg, communication style, visit frequency) to fit local community needs while maintaining the intervention’s core purpose. The functions for the Navigate-Kidney intervention, determined by the community-partnered research team included: (1) build trust through understanding of health experiences; (2) address multi-level social and structural challenges to facilitate health system navigation; (3) provide patient-centered health education; and (4) enhance self-management.

The parallel group, unblinded, patient-level randomized trial of Navigate-Kidney vs standard of care ran from November 2020 August 2022 in 5 urban dialysis centers in Denver, Colorado.^13^ The primary outcome was change in IDWG from 90 days prior to intervention to 180 days postintervention. Secondary outcomes included missed and shortened dialysis sessions, health care utilization, and patient activation.^14^ Patient activation reflects an individual’s knowledge, skills, and confidence in managing their health and health care.^15,16^ The study recruited 139 participants; the majority of whom lived in poverty and reported low-level education and health literacy. Participants had a range of visits from 4 through 19, with a median [IQR] of 6.5 (5.0-8.0) visits. Those randomized to intervention vs standard of care experienced less IDWG with a difference of −0.46% (95% CI, −0.78% to −0.14%), a lower rate per 30 days of shortened dialysis sessions between pre- and postintervention periods of 0.1 (95% CI, −1.2 to 1.1) vs 0.6 (95% CI, −0.5 to 1.8) (*P* = .02), and greater improvement in patient activation with a 4.2 (95% CI, 1.70 to 7.20) difference in the patient activation measure.^13^

Inclusion of patient feedback and input in intervention study design and adaptation to meet participant needs, is a proven strategy to improve intervention fit, and thus, effectiveness.^17,18^ Understanding the experience of receiving the intervention, and how the forms used to achieve the functions were experienced by participants, is therefore critical to ensure sustainment and adaptability of the intervention. We undertook this qualitative study to understand the breadth of participant experiences receiving the Navigate-Kidney intervention.

## Methods

### CHW Training and Activities

Two CHWs were trained and supervised to emphasize the 4 core intervention functions (eTable 1 in Supplement 1), and met the participants biweekly for at least 6 visits. The CHW visits took place primarily in the dialysis center and at home; when visits took place at the dialysis center, the CHW offered to bridge conversations and provide language interpretation when dialysis center clinicians (eg, registered dietitian, social worker, nurse, dialysis technician, or physician) met with the patient.^13^ The CHWs had access to bus tokens, water bottles, and measuring cups which were offered to participants in the Navigate-Kidney intervention group.

### Study Design, Participants, and Settings

Semistructured interviews were conducted with a sample of Navigate-Kidney participants who provided informed consent and received renumeration. Eligible participants were adults (age 18 years or older) with dialysis-dependent kidney failure who self-defined as Latino or Hispanic, participated in and were randomized to the Navigate-Kidney intervention. Participants were sampled from 5 inner-city dialysis centers. Purposive sampling was used to capture a representative proportion of individuals based on food insecurity, sex, and age. We followed the Consolidated Criteria for Reporting Qualitative Research (COREQ) reporting guideline in reporting this study.^19^ The Colorado Multi-Institutional Review Board approved the study.

### Data Collection

The semi-structured interview guide (eTable 2 in Supplement 1) was informed by the Navigate-Kidney intervention functions and forms as well as a literature review regarding barriers and facilitators to CHW interventions.^11,20,21,22,23,24^ Interviews were conducted in-person or by phone in Spanish by 2 Spanish-speaking research assistants who did not have prior interaction with participants. Interviews were continued until thematic saturation, when few or no new concepts were identified in subsequent interviews, was reached.^25^

### Data Analysis

Interviews were audio-recorded, translated to English, transcribed verbatim, deidentified, and imported to qualitative data analysis software Atlas.ti version 9.22 (Scientific Software Development). The transcripts were analyzed by 3 independent coders (A.J, K.R., and J.R.), trained in qualitative research. Using principles of deductive thematic analysis, the interview data were analyzed using a predefined set of themes (Navigate-Kidney functions) to guide coding.^26^ This approach was used to systematically identify and categorize data segments that aligned with existing themes in a theoretical framework to reflect the full range and depth of the data.^27,28,29,30^

We conducted some quantitative analyses from the Navigate-Kidney RCT to understand how the 24 participants compare with the entire sample of participants in the intervention group. First, we compared interviewee baseline characteristics, including key variables, such as demographics, food insecurity, housing stability, and socioeconomic status, stratified by the median observed change in interdialytic weight gain (IDWG). The median cutoff for observed IDWG change was calculated using the full intervention group (N = 68). Interviewees were then classified as either above or below the median for each outcome. Second, primary and secondary outcomes from the quantitative analysis were also reported, stratified by whether intervention participants were interviewed. Data were analyzed from September 2024 to July 2025 using using R software, version 4.4.2 (R Project for Statistical Computing). A 2-sided *P* value of .05 was considered statistically significant.

## Results

Twenty-four Latino participants (11 female [46%], 13 male [54%], mean [SD] age of 56 years [11] and median [range] time on dialysis, 3.5 [2.1-6.0] years) completed interviews (Table 1). Details of the demographics and outcomes are in Table 1 and eTable 2 in Supplement 1. All participants invited agreed to participate in interviews. The median (IQR) length of interviews was 36.2 (28-46) minutes.

**Table 1.** Baseline Characteristics of Interviewed Participants

Characteristic	Participants, No. (%)
Overall intervention, No.	24
Age, mean (SD), y	56.3 (10.7)
Sex	
Female	11 (46)
Male	13 (54)
Self-identified Hispanic or Latino	24 (100)
Self-identified race	
American Indian or Alaska Native	2 (8)
Other or more than 1 race^a^	17 (71)
White	5 (21)
Country of origin	
Mexico	21 (88)
Other (El Salvador, Honduras, Peru)	1 (4)
United States	2 (8)
In general, read and speak language in Spanish	22 (92)
How well do you speak English	
Not at all	5 (21)
Not well	16 (67)
Very well	2 (8)
Well	1 (4)
Highest level of school finished	
High school diploma or GED	3 (12)
Less than high school	18 (75)
More than high school	3 (12)
Current work situation^b^	
Full time work	1 (5)
Otherwise unemployed but not seeking work	19 (86)
Part-time or temporary work	2 (9)
Past year total combined income for you and the family members you live with	
≤$25 000	19 (79)
Don’t know or choose not to answer	1 (4)
>$25 000	4 (17)
Insurance	
Dual Medicare or Medicaid	7 (29)
Medicaid	5 (21)
Medicare	1 (4)
Other public or private	11 (46)
In the past year, have you or any family members you live with been unable to get	
Food	7 (29)
Clothing	7 (29)
Utilities	10 (42)
Child care	2 (8)
Medicine or any health care	11 (46)
Cell phone	7 (29)
In the past 12 mo, how many times did you decide not to fill or refill a prescription because it was too expensive?	
1	2 (8)
2	3 (12)
3-4	3 (12)
None	16 (67)
Mode of transport to and from dialysis	
Benefit transportation (Medicaid)	4 (17)
Family or friend	8 (33)
I drive	6 (25)
Public transportation	6 (25)
Within the past 12 mo, we worried whether our food would run out before we got money to buy more	
Never true	10 (42)
Often true	4 (17)
Sometimes true	10 (42)
Within the past 12 mo, the food we bought just didn’t last and we didn’t have money to get more	
Never true	13 (54)
Often true	4 (17)
Sometimes true	7 (29)
Within the past 12 mo, we couldn’t afford to eat balanced meals	
Never true	8 (33)
Often true	1 (4)
Sometimes true	15 (62)
Worried about losing housing	8 (33)
Patient activation level (PAM)^14^^c^	
1	3 (12)
2	3 (12)
3	7 (29)
4	11 (46)
Months on dialysis, median (IQR)	43.0 (24.8-72.0)
Median interview length, min	36.2

Abbreviations: GED, general education development; PAM, patient activation measure.

^a^ Other race is not further specified to protect participant anonymity.

^b^ Data missing for 2 participants.

^c^ PAM scores categorized into 4 levels (1 to 4) that represent stages of activation—from low activation (level 1: disengaged and overwhelmed) to high activation (level 4: maintaining behaviors and pushing further).

To identify if the participant interviewed were representative of the total participant trial stratified by the median observed change in IDWG (primary outcome), demonstrating relative similarities between groups. Among the 24 interviewees, 10 individuals were above the median observed change in IDWG (eTable 2 in Supplement 1). Second, comparing outcomes between interviewed participants and those who were not interviewed showed that interviewed participants had a greater improvement in the primary outcome, IDWG (median [IQR], −0.82 [−2.84 to −0.07] for interviewees compared with −0.13 [−1.80 to 0.88] for noninterviewees) (Table 2).

**Table 2.** Randomized Clinical Trial Outcomes of Intervention Participants Stratified by Interview Participation (n = 68)

Outcomes	Interviewed (n = 24)	Not interviewed (n = 44)
**Change in PAM Score (follow-up - baseline)^a^**		
Mean (SD)	−0.21 (12.91)	4.19 (13.36)
Median (IQR)	0 (−2.88 to 6.48)	2.20 (−2.12 to 4.78)
**Difference in rate of shortened dialysis sessions per 30 d (post-pre)^b^**		
Mean (SD)	0.66 (1.79)	−0.49 (2.99)
Median (IQR)	0.19 (−0.39 to 1.45)	−0.11 (−1.50 to 1.09)
**Difference in rate of missed dialysis sessions per 30 d (post-pre)^b^**		
Mean (SD)	−0.19 (0.70)	0.14 (0.78)
Median (IQR)	0 (−0.33 to 0)	0 (0 to 0.33)
**Observed change in IDWG (post-pre)^c^**		
Mean (SD)	−1.58 (3.38)	−0.43 (2.19)
Median (IQR)	−0.82 (−2.84 to −0.07)	−0.13 (−1.80 to 0.88)
**Estimated change in IDWG (post-pre)^d^**		
Mean (SD)	−0.41 (0.89)	−0.03 (0.74)
Median (IQR)	−0.29 (−0.77 to 0.13)	0.01 (−0.43 to 0.39)
Median observed change in IDWG (post-pre)^e^		
10 above median, median (IQR)	0.09 (−0.14 to 1.44)	NA
14 below median, median (IQR)	−2.59 (−4.21 to −1.50)	NA

Abbreviations: IDWG, interdialytic weight gain; NA, not available; PAM, patient activation measure.

^a^ PAM scores were calculated as the difference between each participant’s follow-up and baseline scores.

^b^ The rate of missed or shortened dialysis sessions (per 30 days) was defined as the average number of sessions either missed or shortened by 10 minutes or longer, standardized to a 30-day period. Differences were calculated as postintervention minus preintervention rates.

^c^ To account for variability in intervention periods across participants and to align postintervention trajectories, data collected during the intervention period were excluded. Observed changes in IDWG were calculated by subtracting each participant’s first recorded IDWG from their last recorded value.

^d^ To model IDWG over time, we used a precise linear mixed model adjusting for enrollment site, with random intercepts for patients and random slopes for days. We assumed similar preintervention slopes due to randomization. A knot was placed at the start of the intervention period (day 90). Estimated changes in IDWG were calculated similarly to observed changes.

^e^ We stratified the interviewee subgroup based on observed changes in IDWG. Median cutoff for observed IDWG change was calculated using the full intervention group (N = 68): −0.82. Interviewees were then classified as above (n = 10) or below (n = 14) the median for each.

In qualitative interviews, we identified 15 subthemes associated with the 4 intervention functions (Table 3). A schema depicting conceptual links between the themes and subthemes is shown in the Figure.

**Table 3.** Themes and Subthemes by Intervention Function, With Illustrative Patient Statements

Theme (function) and sub themes (forms)	Illustrative statements
**Function 1: Build trust through understanding of health experience**	
Cultivating a personalized connection	I trust the CHW and I invite her to my home. (3)
	She’s [CHW] understanding and caring and feels what you’re going through you know, she’s real good at what she talks, the way she talks to you, you know. (9)
	I trust her so much and I have full confidence in her, she always finds a way to make me feel better. (13)
	Communication with [the CHW] is easier for me because it’s direct communication, just her and me...you feel like you can ask more questions, open up more. (18)
	I observed her (CHW’s) patience. She has this for everyone, no just me, but several others. (45)
	What I really like about her [CHW] is that you feel listened to. (46)
	The greatest support is talking to someone…that there are people who, without knowing you, show interest in your well-being. (56)
Promoting optimism and resilience	She gives me hope. She provides information about what to eat and asks lots of questions so that I understand and I am very happy because she has been kind and given me hope. (3)
	It gave me motivation…she told me, sometimes people don’t have a reason to live, but you have your children, you have your family…And it gave me a lot of motivation to even make dialysis shorter for me. (61)
	She [CHW] gives me hope…she encourages to give life my all, to think about the future.” (11)
	She [CHW] always just tells me “Don’t lose hope.” She’s encouraging me. (9)
	She encourages me to keep going. (26)
	Sometimes you come so burdened with a lot of things…I always think these kinds of practices are good because they’re like therapies, so sometimes they encourage people to keep living, to keep pushing, you know what I mean? (41)
	Well, my self-esteem went up while she was there. (42)
	I’d like to not be passive, to be useful to other people, not just the people who supported me, but to feel useful. (56)
Demonstrating consistency and reliability	She [CHW] says to me, “If you need anything, call me. If you need support or encouragement from someone, you can count on me. Don’t worry…anything like a medication or you feel sad, leave the sadness”….She [CHW] is always there for me. (3)
	She [CHW] is a person, like I was telling you, that gives her time. I know that she is working but not everyone works this hard, she makes time for me and for everyone. I always see her with people, helping. (61)
	Every time I need something, I send her [CHW] a message, like about bills, and she always explains this to me, she explains everything about health insurance…I also have confidence that when I send her a text, because sometimes she doesn’t respond right away, that she will respond. (13)
**Function 2: Address multilevel social and structural challenges to facilitate health system navigation**	
Addressing health insurance barriers	I told [the CHW] I can’t get an identification, because the Mexican Consulate won’t give it to me, and…she said, Let me see how I can help you. (36)
	We ask [the CHW], “Hey, won’t this harm us in any way since we don’t have papers?” “No,” she tells us…and says, “Ask for help, don’t just sit back.” (21)
	[The CHW] helped me with transportation because once they suspended my...all my services because, I hadn’t realized that my residency had expired. (26)
	I was looking for resources to help me—like housing. And they told me I couldn’t apply because I didn’t have papers…[the CHW] she told me: “Don’t worry, I’ll help you look.” (36)
	She helped me with health insurance because I lost my job and so I went from having private health insurance to no insurance. I didn’t have Medicare and she said, “tell me when and we’ll fill out your papers together.” (45)
Improving food security	Since they’d given me the brochures, they explained…what I had to do. We sat there for a long time, like 2 hours, and we talked. Many things, including what to eat, what’s good for me, what’s not. (68)
	She helped me out with resources, there were a couple, food banks too. The food comes monthly and then if I need additional [food] weekly…they give me food there too when I need it…they have good stuff in there like good break I’m able to eat, vegetables and then sometimes they put meat or chicken in there.” (9)
	They send me 6 little boxes with chicken and vegetables. Then there’s a little bag with bread and 2 little boxes of milk, an orange. It’s a big help because I eat that too. It doesn’t have salt; I think that food is very healthy. (3)
	The way the boxes come in is, there’s a little piece of chicken, very finely chopped carrots, peas, and I like them a lot…I haven’t measured it anymore, because I think it’s healthy food. (3)
Relieving the stress of administrative and financial tasks	[The CHW] gave me the cards and the tickets for the bus. (3)
	Since she started, she’s given me bus passes and money for the bill. She’s helped me with the parking ticket with money they put on a card she gave us. (11)
	She tells me, “Call this number and talk to this person,” she says, “They’ll help you,” and I call and I [deidentified] answers, she’s the one I’m talking to. (21)
	She gave me some passes for my bus, which are a great help, because right now, thanks to her, I called the Ride here and got transportation, and they’re taking me to and from dialysis. (33)
	She helped me fill out my insurance, since she saw I was out of work, so my private insurance only covered me for the month…And she told me about Medicare and everything. (45)
Bridging registered dietitian guidance	We worked on communication with the people [dialysis staff] and she supported me, talking especially with the nutritionist on my diet, the types of food I was eating…she [CHW] explained more about what I need to eat, good or bad, what I need to eat to improve my health.
	I had a lot of problems with liquids, I always took too many liquids, and she also took me to see the nutritionist there at the dialysis center and I am better. (3)
	She sometimes helped me with interpretation with the nutritionist. (10)
	We also spoke with the nutritionist so she could check. (17)
	She (CHW) had the nutritionist meet with my daughter and her. (52)
Enabling attendance of and adherence to dialysis sessions and appointments	She gave me my appointments. She said, “look, you have an appointment with Dr [redacted].” She gives me the phone number for the hospital so that I can also make an appointment for my vaccines. I feel so supported by her. I have no one else except my husband. I never had children and so I feel strong support from her. (3)
	She helped me with how to make appointments. (35)
	She also helped me with transportation to get to the Dialysis Center…now that they’re helping me get everything covered, it’s good, right? (17)
	She also told me to go to dialysis and not stop going. (52)
	It motivated me to be a little stricter with everything related to the dialysis rules, because if you break them, someone will sort of pull your ears…and she [CHW] does this kindly and I can’t let her [CHW] down, especially when she is trying to help. (61)
Coordinating nonnephrology clinical care	I wasn’t feeling well and she (CHW) helped me make an appointment with primary care…I have so much to be thankful for. (46)
	She (CHW) called my primary care doctor at my clinic and told my doctor to approve my insulin…because I had gone to my pharmacy and they told me that my doctor had not approved my insulin. That’s when she (CHW) called. (45)
**Function 3: Provide patient-centered education**	
Improving language concordant communication	The CHW speaks English and she interprets for me [in dialysis clinic]..when she is there the language interpretation is better than when they use the screen [ipad] for interpretation. (3)
	She helped me with language interpretation with the registered dietitian. (9)
	When they used the computer interpreter, I didn’t understand well, but when she was there, I understood. (10)
	Until [the CHW] arrived and began to explain everything to me properly in Spanish, I started to get the hang of it. (17)
	It’s easier for me, communicating with [the CHW] because it’s direct communication, just her and me. (18)
	When [de-identified] is helping you, you feel like you can ask more questions, open up more. (18)
	She translated for me sometimes, because I don’t speak English very well. And sometimes when she was there, she translated for me or told the nurses what I needed. (36)
Promoting health and numeracy literacy	She gave me measuring cups…they are little cups made of plastic…so that I could measure my amount of food. I have received so much help from my CHW…I don’t know how to measure and she gave me plastic measuring cups so that I could eat what I measure. One receives so much help from the CHW. It’s because of my CHW that I am still alive and in this clinic. I care about her…She tells me to eat the measurements she gave me. Just eat the measurements from those little plastic pots she gave me. They’re like little cups. She says: just eat the measurements…And that little blue gallon that holds a liter, and what you should drink of water. I have it right there, even full of water, doctor, just like she told me. (3)
	I’m not good with numbers. She [CHW] tells me to record myself telling myself the numbers like the phone number to the dialysis center, the number to transportation, the number of dialysis sessions…(7)
	She gave me a cup and she told me how much I’m able to drink daily. I use that, I’ll fill it up and I’ll use that cup whenever, if I want some water or I put it in the meals I use for the day. (9)
	I only drink water when I take my pills with each meal and my coffee in the morning. I use the bottle that she gave me to subtract the coffee and water that I drink. (11)
	She gave me a beautiful bottle that I learned to measure liquids with. Every time I, for example, drank 8 ounces of coffee…or fruit with juice…or other foods with liquids, I measure it all with the bottle. She [CHW] showed me how to do it. (13)
	I got a water tupperware and I use it to measure the quantity that I drink per day…like juice, everything. (45)
	She gave me a bottle and explained that if I use ice, that needs to go in here…she also told me I can fill it with ice but I also have to use it to measure soups and so it’s 1 L bottle and that’s what I use to measure all liquids. (68)
Providing culturally responsive dietary restriction education	Even though I like beans with cheese…well, I know now how to decrease the cheese and beans I eat and the way that I cook them has also changed. The same with the tomatoes and potatoes. (7)
	First of all, she says, don’t eat so much salt. Because salt is really bad, she says, for your kidneys. She says: so you feel better. Look, here are the portions you should eat. (3)
	She [CHW] tells us, because I had an idea of what foods have potassium…like avocado and tomato…she explains that we can eat them but a smaller amount…balancing the foods that have potassium. (11)
	I never used Mrs. Dash. I found out there was like 8, 9 different types of Mrs. Dash, which was really helpful there because I always thought there was one. (9)
	But she gave me a lot of information, a lot of information about the things I should eat, the liquids I should drink. (11)
	She told me what I should eat, what I shouldn’t eat, because sometimes I saw that my potassium was very high, and she’d tell me what I shouldn’t eat. And then my potassium, what would lower the most, and so on. (42)
	I was initially evaluated and told that I was not a transplant candidate. She [CHW] told me we could get a second opinion. She [CHW] also told me that I would start a diet and exercise and that all of this is possible. (68)
**Function 4: Enhance self-management**	
Exploring opportunity for transplant and peritoneal dialysis	She reminded me about it too: And then, they even have a program it’s a stay home and do your dialysis at home. We could do dialysis and we dialysis all night, all night long while we’re resting and you do it at home here, and she told me about that too. (39)
	I didn’t want to be on the kidney transplant list. I said that’s not for me, no, I didn’t want to be on the list, well. And [the CHW] encouraged me to get on the list, and now I’m on the list. (17)
	No one had talked to me about a transplant even in the 12 y I’ve been on dialysis. The social worker was the first to talk to me about a transplant, about insurance. So if it hadn’t been for [the CHW] you wouldn’t be here right now. (35)
	She (CHW) asked me if I was interested in transplantation and I said, “Of course I am.” (46)
Facilitating quality of dialysis care and symptom management	I don’t like how this person does dialysis because sometimes he doesn’t change his gloves and this is very delicate. And then she spoke with the person in charge of the dialysis center…she actually passed the report directly to the head of dialysis. (41)
	She said, “If they do something else to you again, tell me, because here only the…the sick don’t say anything, that’s why we can’t help them.” (26)
	That’s why when she’s there, I feel safer, because when they’re with me, she tells them, “Leave her here a little longer because she’s bleeding,” and there’s more communication. (18)
	I’ve felt bad on dialysis because they don’t listen to me…When [the CHW] is there interviewing me, if I start to feel bad, I tell her, I’m feeling bad, like my blood pressure is dropping, and she talks to them and they go. (18)
	And the day I went to dialysis, [the CHW] went and I was crying from the pain, because it was too much. She got involved so they could send it to me, the prescription. (36)
	The CHW tells them [dialysis team] that I was feeling bad, that I already had cramps, and they quickly attend to me when CHW was there. (3)
Encouraging medication adherence	She put my phone in MyChart to make my refill of medications at the mail pharmacy. (11)
	She tells me: Take [the medication] like they tell you. Anything you don’t know, just talk to me, she tells me. That’s why I feel her support, a lot of support. (3)
	She showed me how to place my phone number in MyChart so that I could have my medications refilled by the pharmacy by mail…and I tell her that I am very happy. (11)
	She made the application for me and taught me how to order the medications and everything. (35)
	But I would go to the pharmacy [for refill], and they would tell me no, not yet…and then she (CHW) would tell them that they needed to refill my insulin. Then they (pharmacy) would call me back, saying that the medicine was ready and I could go pick it up. (45)
Increasing confidence in diet and fluid self-management	Something I didn’t take into account was when I prepared my food, I didn’t read the labels…She taught me to look at the sodium and potassium on the labels and what’s bad for me. (18)
	What I do like a lot is tomatoes and potatoes, but I try to moderate. Now I do it with a little more confidence after she told me that because I used to eat those foods but with a certain fear of eating them. (11)
	There are things I do now that I didn’t do before. For example, I soak the potato 2 h before cooking it. (13)
	She showed me recipes, and then she told me get on the internet and there was more recipes…And that was real helpful because now I don’t just have to eat just regular rice, I could mix it up with different things. (9)
	Before I used to eat fast food…almost every day…we ate a lot of it and then I started measuring and that’s what really helped us change our diet…this is when I realized we needed to change our diet…now we eat regarly at home and I make my lunch too. (68)
Achieving target clinical parameters	With her help, my phosphorus improved. (42)
	They show me a paper that has faces. When the face is positive or happy, that indicates that your labs like potassium, phosphorus, all of those labs are okay. Lately, I have been getting all positive faces. They [the dialysis clinicians] tell me lately that everything looks good. (7)
	She showed me Mrs. Dash…and my sodium levels are real low, so every month I do a test on me and I get good results out of all of my test now. Before, my results were [not good], now they put little happy faces. (9)
	My blood levels that I have done routinely are getting better and I believe that it’s because of all of her [CHW] advice. (11)
	It seems like an incredible achievement because I arrived 4 or 5 pounds overweight. In fact, I can say that my stomach has gotten smaller with the amount I have to consume because sometimes I eat very little and I feel full, but it’s not that I’m hungry or anything like that, No. (13)
	It was just this sugar that didn’t want to level out, but now it’s levelled out. Now my sugar is fine. I had to give up a lot of things that were sugary. (21)
Activating patients	She does not call to make the appointment. She gives me the phone number and she said, “Look, here’s the phone number.” (3)
	I’m thankful for her [CHW] because sometimes you don’t have people talk to you like that…she encouraged me to get up and do something about my sadness instead of just feeling sorry for myself. (9)
	She gave a lot of guidance about the importance of taking care of oneself…it makes one more responsible and makes one care for oneself more. (56)

Abbrevation: CHW, community health worker.

**Figure.**
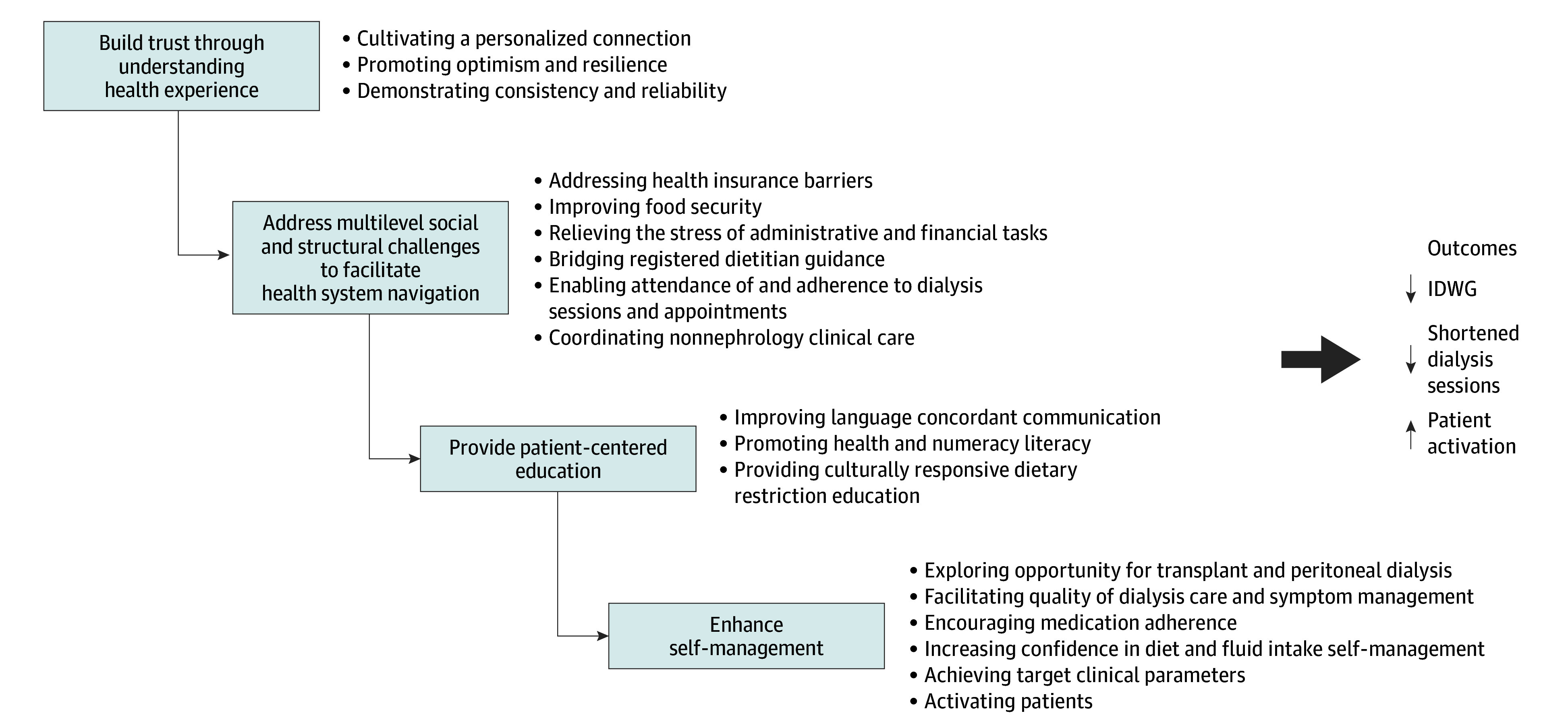
Schema of the Key Relationships Between Themes (Functions) and Subthemes IDWG indicates interdialytic weight gain.

### Function 1: Build Trust Through Understanding of Health Experience

#### Cultivating a Personalized Connection

Participants described that CHWs provided support, demonstrated empathy, and actively listened to their concerns and experiences. One participant noted that this close connection made him feel comfortable, allowing him to share their struggles: “She just kept asking me, ‘Are you okay? Do you need something?’…she made me feel so comfortable that she let me bring it all out.”

#### Promoting Optimism and Resilience

Participants were grateful for the emotional support provided by the CHW, which helped them cope with dialysis: “I learned that I still had a lot of life left to live, even if it is [with dialysis] and that helps me to feel better.” Some participants felt that the opportunity to “vent” to CHWs improved their mental state: “the CHW said, don’t let depression get you…Whatever it is, talk to me.” Participants mentioned that CHWs recognized that they experienced depression, anxiety, and stress, and offered hope and encouragement to access mental health services and social support.

#### Demonstrating Consistency and Reliability

Participants reported that their CHW was responsive and followed through on commitments, including phone calls and visits. They felt they could always rely on their CHW: “The CHW tells me, ‘if you need anything, call me, let me know.’”

### Function 2: Address Multilevel Social and Structural Challenges to Facilitate Health System Navigation

#### Addressing Health Insurance Barriers

Some participants were uncertain about how to obtain insurance and sought advice and support from the CHW. Participants reported the CHW helped them understand insurance options and benefits. The CHW also assisted with social service resources.

#### Improving Food Security

Participants who struggled with food insecurity were provided with resources: “The [CHW] gave me a bag of food every Friday. And they are also helping me with [preparation of] that food.” CHWs helped participants apply for food stamps and enroll in food assistance programs. CHWs also provided education about strategies to purchase affordable ingredients for healthy meals, how to substitute processed or high-salt foods, and taught skills for preparing healthy food.

#### Relieving the Stress of Administrative and Financial Tasks

Participants were overwhelmed by medical and transport expenses, bills for living expenses (eg, electricity and gas bills), employment difficulties during the COVID-19 period, and housing problems. Some participants did not have internet access to apply for benefits; their CHW helped to obtain information and complete benefit applications online. CHWs also helped participants by obtaining financial support (including for transportation), ascertaining what financial benefits participants were entitled to (eg, stimulus payments) and exploring employment opportunities. One participant noted, “The CHW gave me some bus tokens [for dialysis]…It has helped me a lot, because sometimes you don’t even have money for the bus.”

#### Bridging Registered Dietitian Guidance

Participants stated that the CHW joined them when they met with the dialysis center registered dietitian to receive education on nutrition and fluid status: “The nutritionist told me she gave me a paper to understand my diet but when she [CHW] joined us, it was very different…we went over those papers and how the food is related to symptoms…we talked about what to eat and what is good for me and what isn’t good for me.”

#### Enabling Attendance of and Adherence to Dialysis Sessions and Appointments

Participants felt that CHWs helped address barriers to attending medical appointments including arranging transportation. A patient with physical disabilities stated: “[The] CHW took me and walked with me, holding my hand to get to the doctor.” The CHWs arranged transportation for dialysis and participants were “thankful” when they received such help. Participants reported that they felt encouraged to attend dialysis sessions because of the support provided by CHWs during dialysis: “When I started talking to her, I didn’t even realize that it was time to leave dialysis.”

#### Coordinating Nonnephrology Clinical Care

Participants described support from their CHW with medical appointments for dental care, primary care, and for mental health. One participant said, “She helped me with my feet. She [CHW] noticed that my feet looked bad and so she made an appointment for the foot doctor to look at my feet.”

### Function 3: Provide Patient-Centered Education

#### Improving Language Concordant Communication

Participants appreciated having a CHW interpret for them in a clinical setting, which enabled them to comprehend clinician communication: “She helps me understand material or interpret when I go to the dialysis center, because she speaks English and has interpreted for me.” Some participants indicated a preference for their CHW over the center-supplied medical interpreter.

#### Promoting Health and Numeracy Literacy

Participants described not understanding the dietary restriction. Their CHW showed them how to measure and monitor their daily fluid intake using water bottles and measuring cups and one stated “she gave me a water bottle to measure the water...she tells me to only fill this until this line.” Another participant who received measuring cups said, “She (CHW) says, ‘there are ways to measure, how much you can eat and what you can’t.’” CHWs provided participants guidance on food labels and resources such as food-related factsheets and recipes: “My CHW taught me to read, and pay attention to, the labels about sodium in foods.” CHWs also supplied resources, such as food-related fact sheets and recipes.

#### Providing Culturally Responsive Dietary Restriction Education

Participants expressed that CHWs reminded them to limit their sodium intake and helped them understand how to tailor their culturally preferred foods: “The doctors told me not to eat salt but then I spoke to her [CHW] and she explained this, why I should not eat salt, like when I make pico de gallo with tomato and onion.” CHWs helped patients tailor their diet in a way that considered patient preferences: “I didn’t know how to cook certain things…the CHW told me about new menus and recipes.”

### Function 4: Enhance Self-Management

#### Exploring Opportunity for Transplant and Peritoneal Dialysis

Participants reported that CHWs provided participants with information about options for dialysis, including peritoneal dialysis and transplant, and addressed any barriers in access with their clinical team. Some participants learned from and were encouraged by the CHW about the potential for a kidney transplant to improve their health and quality of life. Participants mentioned that the CHW helped them understand and feel supported in their decision-making regarding a kidney transplant decision making and aided in access: “I went to my transplant evaluation by myself, and I was denied. Then I started seeing the CHW and I am now eligible.” The CHWs identified health and financial barriers to transplantation and addressed these with the clinical team.

#### Facilitating Quality of Dialysis Care and Symptom Management

Having a CHW present during dialysis sessions was helpful for participants emotionally and practically: “I feel calmer when the CHW is there [in the dialysis unit].” The CHW provided comfort and helped participants receive better medical care. One participant noted, “They [the staff] leave me for more than 40 minutes holding the little holes of the needles when they take them out…but when the CHW is here and I tell them that I’m ready, they didn’t even take 3 minutes to finish me off [for discharge to home].” Participants believed that CHWs helped them reduce dialysis symptoms and including itching, gastrointestinal problems, cramping, and pain. Some mentioned that CHWs advocated for them and ensured their concerns about symptoms were communicated to and heard by clinicians: “I was crying from the pain in [my] dialysis session and the CHW worked to get me the prescription.”

#### Encouraging Medication Adherence

Participants learned more about their medications from their CHW (eg, “I now know what the medication is for and the function of each medicine”) and realized how the medications could help to improve overall health. CHWs helped them understand the importance of taking their medication “because it can affect my heart or my bones if I don’t take it.” Participants remarked that the CHW assisted with obtaining their medications and getting refills, which prevented them from becoming severely ill: “I would have spent the weekend without any medicine and would have been very sick on Monday due to my kidney problem if the CHWs had not helped me get my medicine.” They provided pill boxes, advised participants about how to prepare their pill boxes, taught participants how to use a mobile application to manage prescriptions, and suggested ways to ensure they had medications available (eg, to bring extra phosphate binders in the car).

#### Increasing Confidence in Diet and Fluid Intake Self-Management

Participants felt motivated by CHWs to change their diet: “The CHW told me how to eat. That if I eat this, everything will turn out well. It motivates me.” Participants mentioned that they were provided with healthier alternatives to salt and better options for oil that they could access in their community. CHWs advised participants on ways to cut down on extra fluid intake, such as taking pills with applesauce instead of water. The CHWs also provided education on potassium and portion control: “One day I was going to eat a banana. No, she told me…Always eat half. Don’t eat it all.” CHWs provided tips on how to limit water intake, such as consuming “sugarless candy, frozen fruit, and ice cubes.”

#### Achieving Target Clinical Parameters

Some participants felt better informed by the CHW about their blood test results (eg, potassium, phosphate) and strategies to meet their target monthly clearance and nutritional parameters. They also better understood and tied their blood tests to symptoms: “the CHW always tells me about my phosphorus and potassium levels. And now that I try to do things the way the CHW suggested, I have felt that my body does feel better.”

#### Activating Patients

Participants described how the CHW worked to activate them by encouraging them to take active steps toward making appointments or asking their clinician for more information. One participant said, “They told me they would call me from the hospital. I didn’t hear. Well, she got me the phone number to contact the hospital to start the transplant [process] and that’s when I started the process.”

## Discussion

These qualitative findings from the Navigate-Kidney trial illustrate that participants experienced the benefits of many activities under each of the core CHW functions of building trust, addressing and navigating the health system, providing education, and enhancing self-management. Through identification of how functions of the intervention were delivered through forms and experienced by participants, this allows for critical and iterative adaptation to ensure that an intervention can fit its context.

Evaluation of how forms and functions are delivered in an intervention can inform policy change by clarifying which elements are essential to achieving desired outcomes and which can be adapted to local contexts. One key aspect is explicitly separating an intervention’s key functions (the essential mechanisms driving outcomes) from its form (the context-specific way those functions are delivered). By making these distinctions, interventions can be evaluated for which components must remain unchanged and which can be adapted, facilitating transferability and scalability without compromising effectiveness. Understanding how these components were experienced by the end user helps policymakers and implementers improve alignment between intervention goals, delivery strategies, and system needs. In the case of dialysis, dialysis organizations are highly motivated to reduce shortened or missed dialysis sessions, given their association with increased mortality, cardiovascular events, and hospitalization. Moreover, the Centers for Medicare & Medicaid Services reimburses CHW activities, contributing to potential sustainment of the intervention. Understanding how the functions and forms were received by participants is therefore critical information when dialysis centers consider using CHW interventions, as it outlines which core elements should remain unchanged vs which forms should be adapted to a local context. By identifying adaptable forms tied to stable functions, policymakers can promote scalable interventions that maintain consistency in purpose while fitting the realities of different communities and resource environments.

Our study indicates that the functions of the intervention were received by participants through the CHW activities. The first function, building trust, underpins effective relationships within health care for Latinos with kidney disease and improves communication and education.^10^ Some of the forms to establishing trust included motivational interviewing with active listening and empathizing and following through on commitments key areas in relationship-building for Latino populations.^31^ Motivational interviewing works by helping individuals resolve ambivalence about change, eliciting their own reasons for change, and supporting autonomy; the addition of motivational interviewing to standard care has been helpful across diverse medical settings.^32,33^ Our CHWs prioritized building trust and addressing social needs early during initial visits, recognizing these as prerequisites for effective self-management education (function 3 and 4), which historically has been limited for Latinos with dialysis-dependent kidney failure.^8,9,10,11^

The second function, addressing and navigating the health system, addressed an identified need by Latino individuals receiving hemodialysis, who have felt they were unable to fully engage in care management due to social challenges.^11^ Similarly, among patients with diabetes, higher levels of social needs were associated with greater perceived stress, problems with executive and cognitive functioning, and less frequent self-care activities.^34^ The addressing social needs, such as transportation, food insecurity, immigration status, financial constraints, and barriers to obtaining insurance.

### Limitations

Our study has limitations. First, it was local to a single community, and our findings may not be generalizable to other settings, though they may be transferable to similar populations. Similarly, there is the possibility of selection bias, as participants with a positive experience may have been more likely to participate. In addition, the Latino cultural context of respeto, which emphasizes respect for authority, harmony, and avoiding confrontation, could have lead to participants giving only favorable responses.^35,36^ In addition, our RCT included 2 CHWs, and our findings may be limited to the individual CHW’s effectiveness in establishing trust with participants. However, we did not note any differences in responses of participants to the 2 CHWs. Finally, as this was a qualitative study and we did not conduct specific analyses of the association of results on different functions to program outcomes, we cannot say that these activities caused the observed improvements.

## Conclusions

In conclusion, this study sheds light on the patient perspective on CHW activities in the Navigate-Kidney intervention. These findings not only affirm the value of CHWs in managing chronic disease but also underscore the importance of adapting the Navigate-Kidney model to other clinical settings and diverse populations, where similar patient-centered approaches may improve outcomes.

## Notes

Supplement 1. **eTable 1. **Forms and functions for Navigate-Kidney CHW intervention **eTable 2.** Interview guide **eTable 3. **Baseline characteristics of interviewed participants based on observed median groupings

Supplement 2.
Data Sharing Statement
