# Supplementary material for: Participant Experiences in a Kidney Failure Care Intervention in the Navigate-Kidney Study
Source: JAMA Netw Open. 2025 Nov 7;8(11):e2548506. doi: 10.1001/jamanetworkopen.2025.48506 (PMC12595532; doi:10.1001/jamanetworkopen.2025.48506)
Supplement: Supplement 1. — eTable 1. Forms and functions for Navigate-Kidney CHW intervention eTable 2. Interview guide eTable 3. Baseline characteristics of interviewed participants based on observed median groupings [file jamanetwopen-e2548506-s001.pdf]

Supplemental Online Content

Rizzolo K, Ressalam J, Robledo K, et al. Participant experiences in kidney failure care intervention: a Navigate-Kidney Study. *JAMA Netw Open*. 2025;8(11):e2548506. doi:10.1001/jamanetworkopen.2025.48506

eTable 1. Forms and Functions for Navigate-Kidney CHW intervention
eTable 2. Interview guide
eTable 3. Baseline Characteristics of Interviewed Participants Based on Observed Median Groupings

This supplemental material has been provided by the authors to give readers additional information about their work.

© 2025 Rizzolo K et al. *JAMA Network Open*.

eTable 1. Forms and Functions for Navigate-Kidney CHW intervention

Functions	Forms
Build trust through understanding of health experience	• Use motivational interviewing: actively listen and empathize<br>• Demonstrate consistency and reliability: follow through on commitments, appointments, and support
Address multi-level social and structural challenges to facilitate health system navigation	• Use social needs screening form<br>• Ask which social need is most distressing/impacting their health<br>• Collaborate with patient to identify practical solutions for top social needs
Provide patient-centered education	• Use motivational interviewing techniques<br>• Explore understanding of kidney failure self care including dietary and fluid restriction<br>• Demonstrate or provide educational materials that are tailored to explain kidney failure, hemodialysis, and dietary restriction
Enhance self-management	• Engage patients using motivational interviewing techniques: asking open-ended questions, reflective listening, affirmations to explore their goals<br>• Encourage active participation in managing kidney care<br>• Guide patients in articulating preferences and concerns<br>• Support patients in discussion with clinicians

© 2025 Rizzolo K et al. *JAMA Network Open*.

eTable 2. Interview guide

1. What thoughts do you have about the peer navigator intervention?
2. What was helpful?
3. What was not helpful?
4. What could be better?
5. Is there something we could do differently?
6. Is there something, about how you take care of yourself or perhaps how you think of your illness, that has changed as a result of this intervention?

© 2025 Rizzolo K et al. *JAMA Network Open*.

eTable 3. Baseline Characteristics of Interviewed Participants Based on Observed Median Groupings (n=24)		
	No. %	
	Above Median[1] (N=10)	Below Median[1] (N=14)
**Age in years, mean (sd)**	56.80 (7.52)	55.86 (12.71)
**Sex**		
Female	4 (40.0%)	7 (50.0%)
Male	6 (60.0%)	7 (50.0%)
**Self-reported Race**		
American Indian/Alaska Native	0 (0%)	2 (14.3%)
White	2 (20.0%)	3 (21.4%)
Other/More than one race	8 (80.0%)	9 (64.3%)
**Country of origin**		
Mexico	9 (90.0%)	12 (85.7%)
USA	0 (0%)	2 (14.3%)
Other (El Salvador, Honduras, Peru)	1 (10.0%)	0 (0%)
**In general, read and speak language other than English**		
Spanish	10 (100%)	12 (85.7%)
English	0 (0%)	2 (14.3%)
**How well do you speak English**		
Not at all	0 (0%)	5 (35.7%)
Not well	9 (90.0%)	7 (50.0%)
Well	1 (10.0%)	0 (0%)
Very Well	0 (0%)	2 (14.3%)
**Highest level of school finished**		
Less than high school	6 (60.0%)	12 (85.7%)
High school diploma or GED	2 (20.0%)	1 (7.1%)
More than high school	2 (20.0%)	1 (7.1%)
**Current work situation**		
Unemployed	0 (0%)	0 (0%)
Part-time or temporary work	1 (10.0%)	1 (7.1%)
Full time work	0 (0%)	1 (7.1%)
Otherwise unemployed but not seeking work	8 (80.0%)	11 (78.6%)
Missing	1 (10.0%)	1 (7.1%)
**Past year total combined income for you and the family members you live with**		
< $25,000	8 (80.0%)	11 (78.6%)
Over $25,000	1 (10.0%)	3 (21.4%)
Don't know/Choose not to answer	1 (10.0%)	0 (0%)
**Insurance**		
Dual Medicare/Medicaid	3 (30.0%)	4 (28.6%)
Medicaid	3 (30.0%)	2 (14.3%)
Medicare	1 (10.0%)	0 (0%)
Other public or Private	3 (30.0%)	8 (57.1%)
**In the past year, have you or any family members you live with been unable to get:**		
Food	4 (40.0%)	3 (21.4%)
Clothing	3 (30.0%)	4 (28.6%)

© 2025 Rizzolo K et al. *JAMA Network Open*.

eTable 3. Baseline Characteristics of Interviewed Participants Based on Observed Median Groupings (n=24)		
	No. %	
	Above Median[1] (N=10)	Below Median[1] (N=14)
Utilities	5 (50.0%)	5 (35.7%)
Child care	0 (0%)	2 (14.3%)
Medicine or any healthcare	5 (50.0%)	6 (42.9%)
Cell Phone	3 (30.0%)	4 (28.6%)
**In the past 12 months, how many times did you decide not to fill or refill a prescription because it was too expensive?**		
None	6 (60.0%)	10 (71.4%)
1 time	2 (20.0%)	0 (0%)
2 times	1 (10.0%)	2 (14.3%)
3-4 times	1 (10.0%)	2 (14.3%)
**Mode of transport to and from dialysis**		
Benefit transportation (Medicaid)	2 (20.0%)	2 (14.3%)
Family or friend	2 (20.0%)	6 (42.9%)
I drive	2 (20.0%)	4 (28.6%)
Public transportation	4 (40.0%)	2 (14.3%)
**Within the past 12 months, we worried whether our food would run out before we got money to buy more**		
Never true	4 (40.0%)	6 (42.9%)
Sometimes true	5 (50.0%)	5 (35.7%)
Often true	1 (10.0%)	3 (21.4%)
**Within the past 12 months, the food we bought just didn't last and we didn't have money to get more**		
Never true	6 (60.0%)	7 (50.0%)
Sometimes true	3 (30.0%)	4 (28.6%)
Often true	1 (10.0%)	3 (21.4%)
**Within the past 12 months, we couldn't afford to eat balanced meals**		
Never true	3 (30.0%)	5 (35.7%)
Sometimes true	6 (60.0%)	9 (64.3%)
Often true	1 (10.0%)	0 (0%)
**Are you worried about losing your housing?**		
No	6 (60.0%)	10 (71.4%)
Yes	4 (40.0%)	4 (28.6%)
**Patient Activation Level (PAM)**		
1	0 (0%)	3 (21.4%)
2	2 (20.0%)	1 (7.1%)
3	2 (20.0%)	5 (35.7%)
4	6 (60.0%)	5 (35.7%)
**Months on dialysis, median (IQR)**	35.00 (26.25, 43.50)	57.00 (27.75, 81.00)
**Total number of CHW visits**		
Mean (SD)	6.60 (2.37)	7.21 (2.19)
Median (IQR)	5.00 (5.00, 8.75)	6.50 (6.00, 8.00)
**Duration of interview in minutes**		

© 2025 Rizzolo K et al. *JAMA Network Open*.

eTable 3. Baseline Characteristics of Interviewed Participants Based on Observed Median Groupings (n=24)

	No. %	
	Above Median[1] (N=10)	Below Median[1] (N=14)
Mean (SD)	35.70 (11.88)	37.00 (14.46)
Median (IQR)	34.00 (27.50, 42.25)	40.00 (29.50, 46.00)

[1] The interviewee stratification is based on the median cutoff for observed Interdialytic Weight Gain (IDWG). The median cutoff for observed IDWG change was calculated using the full intervention group (N = 68), with an observed median change of −0.82. Interviewees were then classified as above or below the median.

© 2025 Rizzolo K et al. *JAMA Network Open*.
