# Supplementary material for: Participant Experiences in a Kidney Failure Care Intervention in the Navigate-Kidney Study
Source: JAMA Netw Open. 2025 Nov 7;8(11):e2548506. doi: 10.1001/jamanetworkopen.2025.48506 (PMC12595532; doi:10.1001/jamanetworkopen.2025.48506)
Supplement: Supplement 2. — Data Sharing Statement [file jamanetwopen-e2548506-s002.pdf]

Data Sharing Statement

Data
Data available: No

Additional Information
Explanation for why data not available: The data are qualitative interview transcripts.
